# Case Report: Systemic Small-Vessel Vasculitis in an Adolescent With Active Ulcerative Colitis

**DOI:** 10.3389/fped.2021.617312

**Published:** 2021-02-10

**Authors:** Marleen Bouhuys, Wineke Armbrust, Patrick F. van Rheenen

**Affiliations:** ^1^Department of Pediatric Gastroenterology, Hepatology and Nutrition, Beatrix Children's Hospital, University Medical Center Groningen, University of Groningen, Groningen, Netherlands; ^2^Department of Pediatric Rheumatology and Immunology, Beatrix Children's Hospital, University Medical Center Groningen, University of Groningen, Groningen, Netherlands

**Keywords:** inflammatory bowel diseases, colitis ulcerative, systemic vasculitis, anti-neutrophil cytoplasmic antibody-associated vasculitis, purpura

## Abstract

**Introduction:** Small-vessel vasculitis (SVV) is a rare immunological disease that affects arterioles, capillaries and venules. It causes purpura, but can also manifest in other organs, including the gastrointestinal tract. SVV and inflammatory bowel disease (IBD) co-occur more frequently than would be expected by chance.

**Case description:** A 16-year-old girl, who had been diagnosed with ulcerative colitis (UC) 2 years earlier at a general hospital, developed purpura, progressive abdominal pain with frequent bloody diarrhea and frontotemporal headache and swelling while on azathioprine and mesalamine maintenance therapy. Serology was positive for perinuclear antineutrophil cytoplasmic antibodies (p-ANCA) without antiprotease- or myeloperoixidase antibodies. Endoscopy revealed active left-sided UC and atypical ulcerations in the ascending colon. Biopsies of these ulcerations and of affected skin revealed leukocytoclastic vasculitis. Initially this was interpreted as an extraintestinal manifestation of UC that would subside when remission was induced, consequently infliximab was started. Over the next 3 weeks she developed severe burning pain in her right lower leg that progressed to a foot drop with numbness and the purpura progressed to bullous lesions. The diagnosis was adjusted to ANCA-associated vasculitis with involvement of skin, bowel and peripheral nerves. Infliximab was discontinued and induction treatment with high-dose prednisolone and cyclophosphamide was given until remission of SVV and UC was achieved. Subsequently, infliximab induction and maintenance was re-introduced in combination with methotrexate. Remission has been maintained successfully for over 2 years now. The foot drop only partly resolved and necessitated the use of an orthosis.

**Conclusion:** Pediatric patients with IBD who present with purpuric skin lesions and abdominal pain should be evaluated for systemic involvement of SVV, which includes endoscopic evaluation of the gastrointestinal tract. We discuss a practical approach to the diagnosis, evaluation and management of systemic SVV with a focus on prompt recognition and early aggressive therapy to improve outcome.

## Introduction

Vasculitis comprises a heterogeneous group of disorders in which inflammation of blood vessel walls is present. Epidemiological data on childhood vasculitis is scarce and poorly characterized. The estimated overall annual incidence rate of primary childhood vasculitis is 22.8 per 100,000, but this includes the relatively common childhood vasculitides Henoch Schönlein purpura (HSP) and Kawasaki disease (1). When these types, with their typical pattern of (muco)cutaneous inflammation, are disregarded, the incidence rate drops to 0.24 per 100,000. Due to its rarity and broad spectrum of clinical presentations, diagnosis of childhood vasculitis is often delayed, leading to significant morbidity and mortality (2–4).

According to the internationally accepted 2012 Chapel Hill Consensus Conference nomenclature, vasculitis is defined by histopathology, immunological findings and, above all, the size of the predominantly affected vessel (i.e., small, medium, or large) (5–7). Small vessel vasculitis (SVV) is characterized by inflammation of parenchymal arteries, arterioles, capillaries and venules and is subdivided into antineutrophil cytoplasmic antibody (ANCA)-associated vasculitis and immune-complex-mediated vasculitis (including HSP) (7).

Some patients with SVV are originally diagnosed as having single-organ involvement (e.g., cutaneous SVV), but many will develop additional disease manifestations that warrant redefining the case as a multi-organ SVV.

Inflammatory bowel disease (IBD) and SVV co-occur more often than would be expected by chance (8–10). Additionally, SVV itself can also present with gastrointestinal symptoms. When the small or medium vessels of the gastrointestinal tract are involved, patients can present with symptoms ranging from mild abdominal pain and diarrhea to life-threatening colonic ischemic ulcers, peritonitis and bowel perforations (11, 12). In patients who were previously diagnosed with IBD, distinguishing between these two clinical entities can be challenging. Increased fecal calprotectin levels are found in both active IBD and SVV with bowel involvement and are therefore not indicative (13, 14).

## Case Description

**Figure 1:**
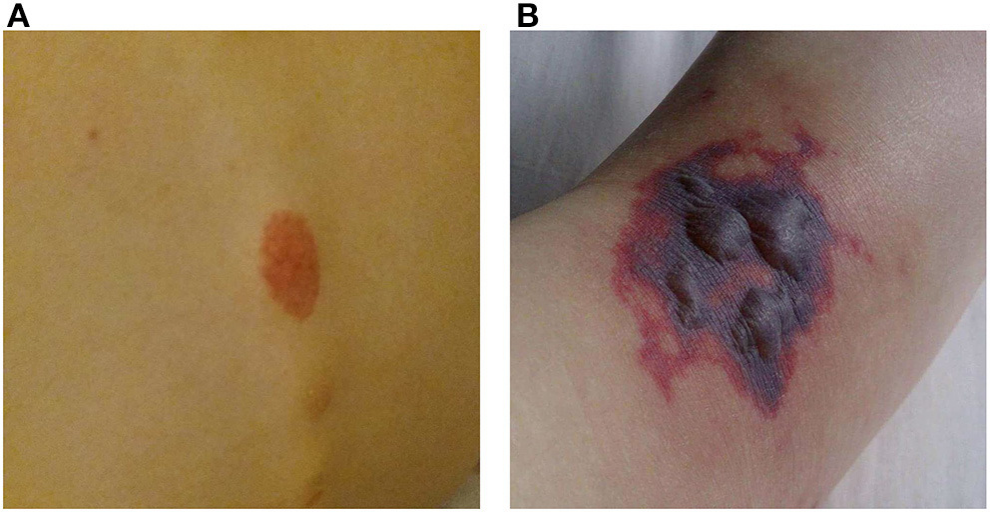
Purpura. **(A)** Purpura located on the lower back. **(B)** Purpura with blistering located on the dorsal side of the right foot.

A 16-year-old girl, who had been diagnosed with ulcerative colitis (UC) 2 years earlier at a general hospital, recently had several flares of UC despite treatment with azathioprine, mesalamine, and several tapering courses of corticosteroids. She was referred to our University Hospital for a second opinion with respect to a possible step-up to anti-TNF therapy. In the week prior to colonoscopy the patient developed painful purpura on the lower leg (causing an antalgic gait) and dorsal trunk. One day prior to the planned colonoscopy she was admitted to our hospital with progressive abdominal pain and frequent bloody diarrhea. Some of the purpura had faded while new ones had appeared (Figure 1A). Peripheral blood analysis revealed increased C-reactive protein (CRP 71 mg/L; normal range <5 mg/L) and erythrocyte sedimentation rate (71 mm/h; normal range <20 mm/h). Coagulation tests, creatinine, aspartate aminotransferase, alanine aminotransferase and gamma-glutamyl transferase were within normal limits. Fecal calprotectin was 4,010 μg/g (target range <150 μg/g). Urinalysis was normal.

**Figure 2:**
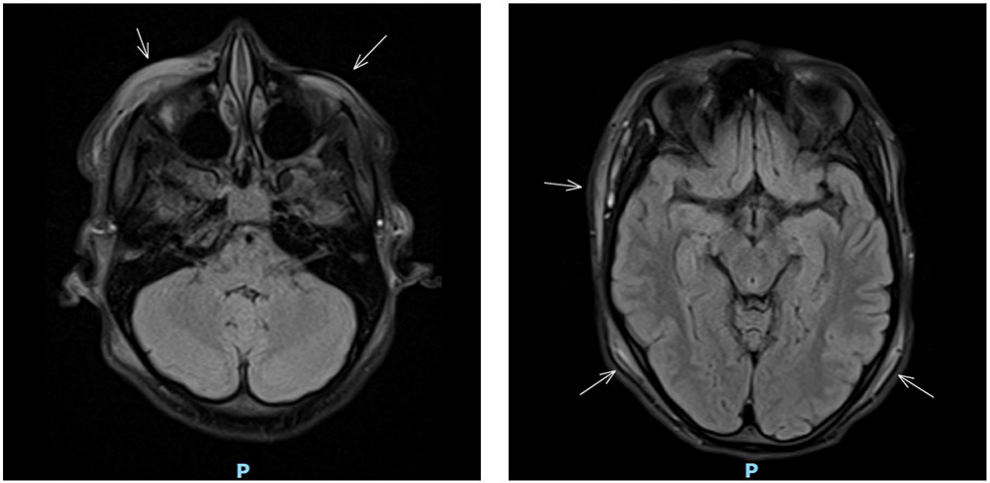
Cranial MRI showing diffuse subcutaneous edema (indicated by arrows).

That night the girl woke up with excruciating right temporal headache similar to the pain she experienced a week before at the site of the purpura. The headache was accompanied by frontotemporal swelling. A cranial MRI was performed, which showed diffuse subcutaneous edema without intracranial pathology (Figure 2).

**Figure 3:**
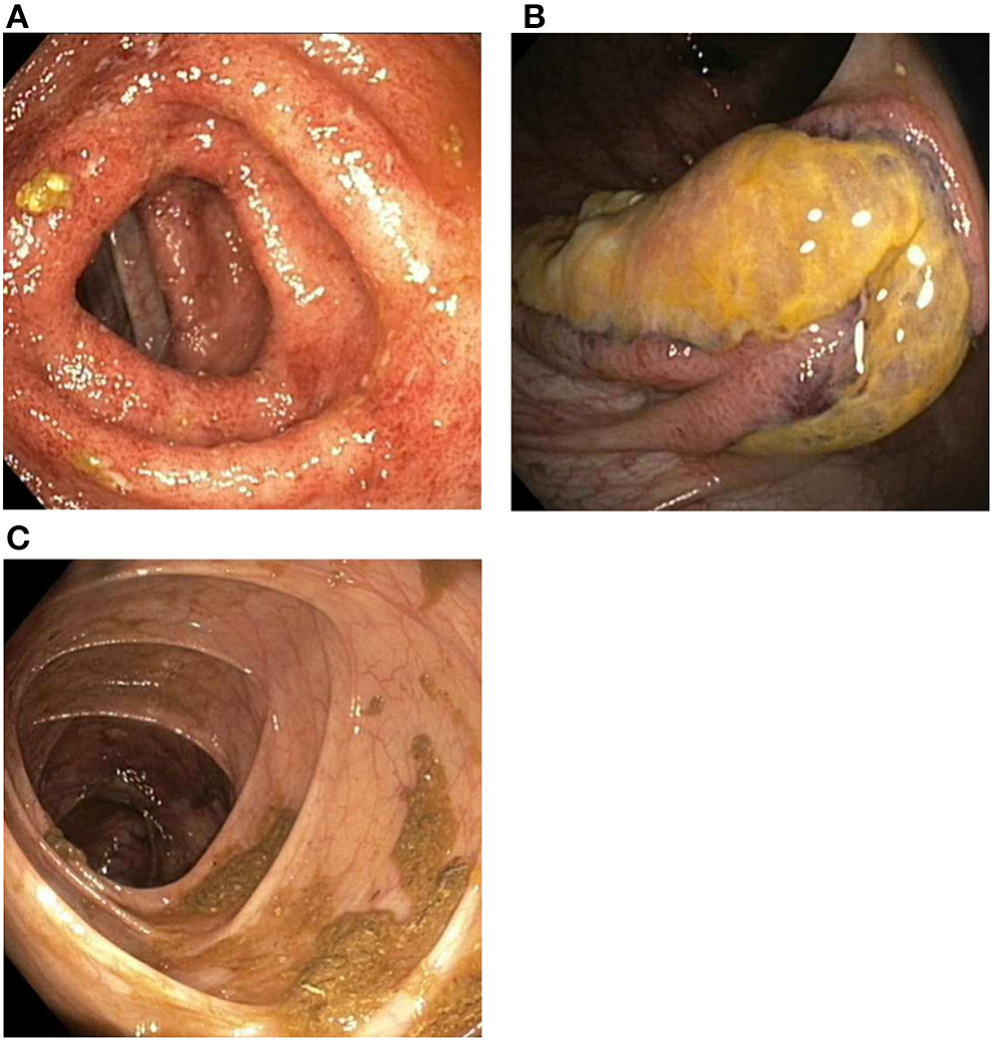
Findings during colonoscopic evaluation. **(A)** Inflammation of the transverse colon, including erythema, a granular appearance, friability and loss of the normal fine vascular pattern. **(B)** Ulcerations located in the ascending colon. **(C)** Healed ascending colon after 5 weeks of chemotherapy.

The next day, colonoscopic examination showed continuous inflammation in the left transverse (Figure 3A), descending and sigmoid colon with rectal sparing, suggestive of active UC. In the ascending colon three atypical ulcerations were seen (Figure 3B). Histological examination of these ulcerations showed necrosis of the mucosa and submucosa with thrombosis and perivascular infiltrates with neutrophils and nuclear debris, consistent with leukocytoclastic vasculitis. There were no granulomatous changes or immune deposits. Biopsies of the terminal ileum, cecum, right transverse colon and rectum showed no abnormalities. The descending and sigmoid colon showed a histological pattern of chronic inflammation defined by crypt architectural distortion and basal lymphoplasmacytosis. A punch biopsy from the skin of the left lower leg, obtained during the colonoscopic session, showed perivascular infiltrates with neutrophils in the dermis, with affected vessel walls and extravasation of erythrocytes, and was also consistent with leukocytoclastic vasculitis. Immunofluorescence was negative. Serum perinuclear (p)-ANCA was positive (titer >1:640) without antiprotease- or myeloperoxidase antibodies. Rheumatoid factor, anticardiolipin antibodies, cryoglobulins and antinuclear antibodies were negative. Neither did we find antibodies against hepatitis A and B virus, cytomegalovirus and Epstein-Barr virus. Chest X-ray was normal.

It was reasoned that the increased serological and fecal markers of inflammation and leukocytoclastic vasculitis were manifestations of active UC, and that these symptoms would subside upon reaching remission. Positivity for p-ANCA is observed in the majority of cases of UC and was interpreted as an immune response associated with the disease process itself. Intravenous administration of infliximab 5 mg/kg was planned with three induction doses over 6 weeks (week 0-2-6), followed by maintenance therapy every 8 weeks. Azathioprine and mesalamine maintenance therapy was continued.

The frontotemporal subcutaneous edema resolved, but after the second infliximab administration fever appeared, CRP gradually rose to 230 mg/L, the purpura progressed to purple blisters (Figure 1B) and the patient developed a progressive paralysis of the right lower leg, resulting in a complete foot drop. Neurological examination revealed absence of the Achilles stretch reflex and plantar reflex on the right side, and sensory disturbances on the right foot and left hand. Electromyography showed active axonal damage of the left median nerve and the right peroneal and tibial nerve (i.e., multiple peripheral mononeuropathy). Spinal MRI was normal.

**Figure 4:**
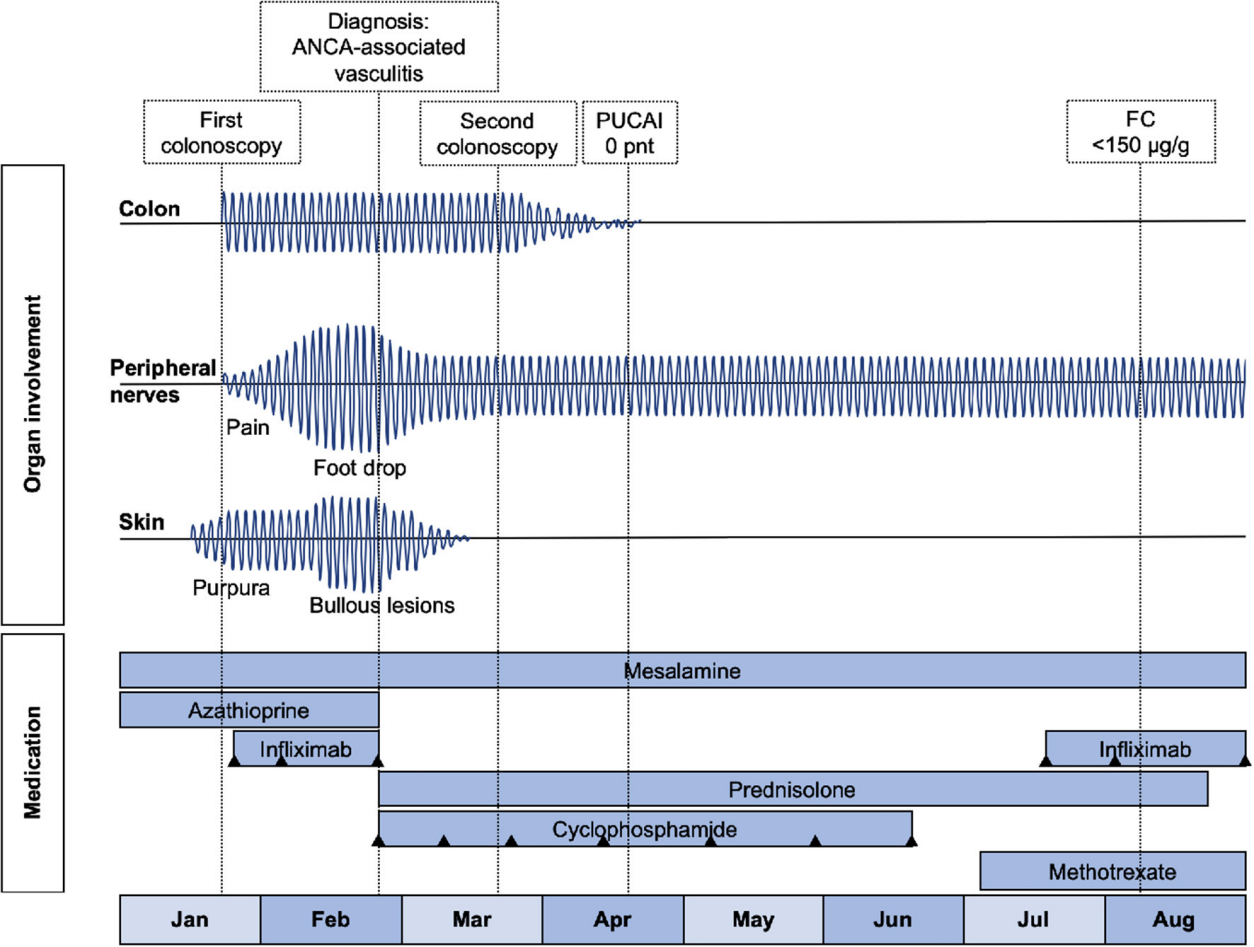
Timeline showing disease intensity per organ and medication. The black triangles mark the infusions. PUCAI, Pediatric Ulcerative Colitis Activity Index; FC, Fecal calprotectin.

The multitude of clinical signs (blistering purpura, mononeuritis multiplex, fever and involvement of the gastro-intestinal tract), in combination with histopathology (leukocytoclastic vasculitis without immune deposits) and immunological findings (p-ANCA positivity and high CRP), made us reconsider our initial frame of mind. We ascertained the diagnosis as ANCA-associated vasculitis (subclassified as microscopic polyangiitis), with damage of the small blood vessels in skin, colon and peripheral nerves. Because of the severe presentation, induction therapy was started consisting of high-dose intravenous prednisolone (60 mg/day) and intravenous cyclophosphamide (15 mg/kg at week 0-2-4, followed by once every 3 weeks until 3 months of stable remission). Infliximab and azathioprine were discontinued. As a result, UC treatment was de-escalated to mesalamine monotherapy (Figure 4).

Remission of cutaneous and colonic vasculitis (Figure 3C) and normalization of CRP were achieved after 5 weeks of chemotherapy. Prednisolone was tapered and stopped after 4 months and cyclophosphamide was discontinued after seven infusions. Infliximab was re-introduced with three induction doses (5 mg/kg) over 6 weeks, after which fecal calprotectin had declined to the target range (77 μg/g). Before the fourth infusion we measured a suboptimal infliximab trough level and consequently escalated to 10 mg/kg every 8 weeks. All subsequent trough levels were in range (i.e., ≥5 μg/ml). Oral methotrexate 20 mg once weekly was started to reduce the risk for recurrence of vasculitis. Biochemical and clinical remission of both ANCA-associated vasculitis and ulcerative colitis have been maintained successfully for over 2 years now, with persistent normal CRP and fecal calprotectin levels. Neurological sequelae including a partial foot drop (necessitating the use of an orthosis) and neuropathic pain remain.

## Discussion

We present the case of a 16-year-old girl with active UC complicated by systemic ANCA-associated SVV, resulting in neurological damage. In ANCA-associated SVV, systemic involvement is common, which necessitates a structured organ-based medical history and physical examination in all patients, even when they present with apparently isolated skin lesions. Use of a clinical tool such as the Birmingham Vasculitis Activity Score (15) and the Five Factor Score (16) help the physician with a structured clinical assessment. ANCA testing and a skin punch biopsy with immunofluorescence should be performed to help confirm and (sub)classify SVV. Organ-oriented laboratory panels (such as urinalysis, serum creatinine and blood urea nitrogen for kidney function), imaging and tissue sampling may aid to diagnose systemic SVV (17, 18). In IBD patients with suspected vasculitis and gastrointestinal symptoms, this implies endoscopic evaluation to distinguish active IBD from SVV with bowel involvement.

### Why Did we Initially Miss the Diagnosis?

We had histological proof of involvement of vasculitis in two organs (skin and bowel) at an early stage, yet we failed to link the serological test results to ANCA-associated vasculitis. In our patient p-ANCA positivity was observed in the absence of antiprotease- and myeloperoxidase antibodies. This pattern is called atypical p-ANCA and is frequently seen in patients with UC (19), while ANCA-associated SVV in IBD is extremely rare.

The notion that the vasculitis in our patient was an extraintestinal manifestation of IBD that would subside after treatment with intravenous infliximab was incorrect. Although erythema nodosum, a well-recognized extra-intestinal manifestations of IBD, usually resolves with treatment of the underlying IBD (20), this is not the case for IBD-associated vasculitis. This misconception contributed to a serious delay in adequate treatment. Another sign that should have raised suspicion toward severe systemic vasculitis was the exceptionally high CRP, whereas, in active UC the CRP response is usually limited (21).

### Why Is Early Recognition Important?

Peripheral nerve damage was recognized only after the patient developed a complete foot drop, while in retrospect, an abnormal gait had already been observed during the first clinical assessment when it was thought to be caused by the painful skin lesions. In fact, when purpura and excruciating pain develop concurrently, the index of suspicion for SVV, in particular ANCA-associated vasculitis should be high. Failure to recognize systemic involvement at an early stage delays treatment, increases organ damage and decreases quality of life (22). In an international cohort study consisting of 105 patients with childhood-onset ANCA-associated vasculitis, evidence of damage was still present in over 60% of patients 12 months after diagnosis (23). In a French nationwide cohort study kidney damage was most frequently seen with 34% of patients developing end-stage renal disease after a median follow-up of 5.2 years (24). We feel that reducing the time before consulting a specialized (pediatric) immunologist and instituting early aggressive therapy will probably improve patient outcome.

### How to Treat Systemic SVV?

Because of its rarity, specific guidelines on the management of systemic vasculitis in children are lacking. To fill this gap, the European initiative Single Hub and Access point for pediatric Rheumatology in Europe (SHARE) developed best practice recommendations (18). In addition, The European League Against Rheumatism, the European Renal Association and the European Vasculitis Society have published recommendations for the management of ANCA-associated vasculitis in adults (25). Our patient was ultimately treated with high-dose prednisolone and intravenous cyclophosphamide. This is in accordance with the SHARE recommendations, that advise prednisolone to be dosed at 1–2 mg/kg/day, to a maximum of 60 mg/day and cyclophosphamide every 3 weeks at 500–1,000 mg/m2 to a maximum of 1.2 g. Rituximab (especially in ANCA-associated vasculitis), methotrexate or mycophenolate mofetil (MMF) can also be used as induction therapy, always in combination with high-dose prednisolone. We chose pulse intravenous cyclophosphamide as there is anecdotal evidence that it also has a remission-inducing effect in UC (26, 27). Usually, induction therapy is continued for 3–6 months and prednisolone is tapered during this period. First-line maintenance therapeutic agents recommended by SHARE are azathioprine or MMF. Second-line maintenance therapy includes methotrexate and rituximab. In our patient who suffered from both SVV and UC, maintenance therapy consisted of infliximab and methotrexate. Azathioprine immunomodulation would have been a more logical choice, but we followed the patient's personal preference based on earlier experienced side effects. Once-weekly oral methotrexate administered concomitantly with infliximab is known to reduce the likelihood of anti-infliximab antibody development and the associated secondary loss of response (28). The common duration of maintenance therapy for SVV is about 1–1.5 year. Stopping methotrexate could be considered when infliximab trough levels are in the target range and endoscopic healing is achieved.

There are numerous reports that anti-TNF therapy by itself increases the risk of vasculitis, in particular the immune-complex-mediated type including HSP (29, 30). The association between anti-TNF and HSP is supported by a resolution of vasculitis when the biological agent is discontinued, and by reappearance of symptoms during a rechallenge. In our patient SVV manifested before the onset of anti-TNF therapy, which makes a causal relation highly unlikely, although we cannot exclude that infliximab might have played a role in the aggravation of the vasculitis. We were of the opinion that in this particular case re-introduction of infliximab was a safe option to maintain remission in UC. If vasculitis manifests in a patient receiving anti-TNF therapy, an association between the two should be considered and, if possible, excluded. In case of a (suspected) association, biological therapy with a different class of agent (such as vedolizumab or ustekinumab) could be considered.

This case illustrates the difficulties and misconceptions that can be encountered in the diagnosis and treatment of SVV in the pediatric age group. Prompt recognition and early aggressive therapy are key in order to prevent morbidity and mortality. If induction therapy would have been started earlier, the neurological sequelae in our patient could have been prevented.

## Patient Perspective

“When the first skin lesions appeared, I did not think much of it, but from then on new lesions appeared almost every day. It always started with a terrible burning pain, followed by wounds and swellings, the scars of which are still visible. In the beginning I felt my complaints were waved aside, which was very frustrating. It was said that things would get better when my ulcerative colitis would calm down, but at the same time my condition deteriorated quickly. I had the feeling that something serious was going on. When my nerves stopped working and my entire foot became paralyzed the vasculitis was finally recognized and dealt with. It was a bit of a shock to hear that I had to have chemotherapy, but after the first infusion I could already tell that my symptoms were getting better. Compared to the symptoms I experienced earlier, the treatment was not a big deal and I found a lot of comfort in talking with my doctor. Looking back, I think it is regrettable that treatment was not started earlier, maybe then I would not have had the nerve damage that causes pain and other struggles up to this day.”

## Data Availability Statement

The original contributions presented in the study are included in the article/supplementary material, further inquiries can be directed to the corresponding author/s.

## Ethics Statement

Ethical review and approval was not required for the study on human participants in accordance with the local legislation and institutional requirements. Written informed consent was obtained from the individual for the publication of any potentially identifiable images or data included in this article.

## Author Contributions

MB drafted and revised the manuscript and approved the final manuscript as submitted. PR drafted, reviewed and revised the manuscript, and approved the final manuscript as submitted. WA reviewed and revised the manuscript, and approved the final manuscript as submitted. All authors approved the final manuscript as submitted and agree to be accountable for all aspects of the work.

## Conflict of Interest

The authors declare that the research was conducted in the absence of any commercial or financial relationships that could be construed as a potential conflict of interest.
